# Dysfunctional Brain Dynamics of Parkinson's Disease and the Effect of Acute Deep Brain Stimulation

**DOI:** 10.3389/fnins.2021.697909

**Published:** 2021-07-20

**Authors:** Zhibao Li, Guoping Ren, Chong Liu, Qiao Wang, Kun Liang, Chunlei Han, Hui Qiao, Jianguo Zhang, Qun Wang, Fangang Meng

**Affiliations:** ^1^Department of Functional Neurosurgery, Beijing Neurosurgical Institute, Capital Medical University, Beijing, China; ^2^Department of Neurology, Beijing Tiantan Hospital, Capital Medical University, Beijing, China; ^3^China National Clinical Research Center for Neurological Diseases, Beijing, China; ^4^Department of Functional Neurosurgery, Beijing Tiantan Hospital, Capital Medical University, Beijing, China; ^5^Beijing Key Laboratory of Neurostimulation, Beijing Neurosurgical Institute, Beijing, China; ^6^Neuroelectrophysiology Room, Beijing Neurosurgical Institute, Capital Medical University, Beijing, China; ^7^Chinese Institute for Brain Research Beijing (CIBR), Beijing, China

**Keywords:** Parkinson's disease, EEG microstate, brain dynamic, deep brain stimulation, brain network, resting state

## Abstract

**Background:** Parkinson's disease (PD) is the second most common neurodegenerative disorder after Alzheimer's disease, and deep brain stimulation (DBS) can effectively alleviate PD symptoms. Although previous studies have detected network features of PD and DBS, few studies have considered their dynamic characteristics.

**Objective:** We tested two hypotheses. (1) Reduced brain dynamics, as evidenced by slowed microstate dynamic change, is a characteristic of PD and is related to the movement disorders of patients with PD. (2) Therapeutic acute DBS can partially reverse slow brain dynamics in PD to healthy levels.

**Methods:** We used electroencephalography (EEG) microstate analysis based on high density (256-channel) EEG to detect the effects of PD and DBS on brain dynamic changes on a sub-second timescale. We compared 21 healthy controls (HCs) with 20 patients with PD who were in either DBS-OFF or DBS-ON states. Assessment of movement disorder using the Unified Parkinson's Disease Rating Scale III was correlated with microstate parameters.

**Results:** Compared with HCs, patients with PD displayed a longer mean microstate duration with reduced occurrence per second, which were significantly associated with movement disorders. In patients with PD, some parameters of microstate analysis were restored toward healthy levels after DBS.

**Conclusions:** Resting-state EEG microstate analysis is an important tool for investigating brain dynamic changes in PD and DBS. PD can slow down brain dynamic change, and therapeutic acute DBS can partially reverse this change toward a healthy level.

## Introduction

Parkinson's disease (PD) is the second most common neurodegenerative disorder after Alzheimer's disease (Reich and Savitt, 2019). It is characterized by a series of motor symptoms, such as tremor, stiffness, slowness, and imbalance, and various non-motor (non-movement) symptoms, such as depression, sleep disturbances, and dementia (Armstrong and Okun, 2020). PD is mainly caused by the death of dopaminergic neurons in the substantia nigra. This results in dopamine deficiency in mesencephalic structures and the basal ganglia, which subsequently affects the neocortex (Jucker and Walker, 2013; Armstrong and Okun, 2020). Large-scale and distributed network changes caused by this pathological mechanism have been demonstrated by numerous studies (Oswal et al., 2016; Kim et al., 2017; Ma et al., 2017).

Impaired functional connectivity in the cortical-striatal loop and related neural circuits have been observed in PD by resting-state functional MRI (fMRI) (Helmich et al., 2010; Hacker et al., 2012). However, other findings indicate that the effects of PD may not only be limited to cortical-striatal loop impairments but might also involve whole-brain functional networks. Several studies have shown that PD induces abnormal topology of brain functional connectivity within the triple network model [the default mode network (DMN), the salience network (SN), and the executive/frontoparietal network (FPN)] and in other networks, including motor and visual networks (Skidmore et al., 2011; Baggio et al., 2014; Lebedev et al., 2014; Gorges et al., 2015; Luo et al., 2015; Putcha et al., 2015; Tinaz et al., 2016). Large-scale brain network analysis based on the graph theory approaches has shown abnormal topological characteristics in the brain network, which help in identifying and tracking PD (Skidmore et al., 2011; Gottlich et al., 2013; Luo et al., 2015). More specifically, the brain network of patients with PD has abnormal local and global efficiency of parallel information transfer. Overall, the brain network abnormalities in patients with PD are not local or limited to several neural circuits but are large scale and distributed.

Deep brain stimulation (DBS) is the most effective surgical treatment in the middle and advanced stages of the disease, and several mechanisms of action have been proposed in recent decades (Lozano and Lipsman, 2013). Gradually, treatment and research paradigms have shifted away from localized stimulation of specific brain nuclei toward global modulation of large-scale brain networks (McIntyre and Hahn, 2010; Litvak et al., 2011; Vanegas-Arroyave et al., 2016; Akram et al., 2017; Horn et al., 2017, 2019; Shen et al., 2020). Oswal et al. (2016) used magnetoencephalography and local field potential recording to demonstrate that the therapeutic effects of DBS in patients with PD were indeed selective attenuation of the presumed hyper-direct drive to the subthalamic nucleus (STN) and suppression of the low beta oscillatory range in the STN. Furthermore, a recent study has demonstrated that STN-DBS modulates two distinct neurocircuits, the GPi-thalamus-deep cerebellar nuclei circuit and the M1-putamen-cerebellum circuit (Shen et al., 2020). STN-DBS can activate the former to improve movement and inhibit the latter to improve bradykinesia. In addition, another study has shown that DBS can increase overall connectivity in the motor network, normalize the network profile toward that of healthy controls (HCs), and specifically enhance thalamocortical connectivity while diminishing striatal control over basal ganglia and cerebellar structures. Therefore, DBS has a systemic impact.

Although previous studies have explored the mechanisms of DBS in PD with a network view, few studies have considered the dynamic characteristics of the brain (Kim et al., 2017). Furthermore, most previous studies detected brain dynamics by fMRI, which has a low temporal resolution. Electroencephalography (EEG), however, has a high temporal resolution and can detect dynamic changes in the brain on a sub-second timescale (Michel and Koenig, 2018). EEG is not consistent but can be divided into several non-overlapping, quasi-stable topographies. These topographies, termed EEG microstates, usually remain transiently stable for around 80–120 ms and then abruptly transform into a new state (Khanna et al., 2015; Michel and Koenig, 2018). Even though many microstates are evident in EEG, in clinical research, four microstate classes are usually used (Koenig et al., 1999; Britz et al., 2010; Brodbeck et al., 2012). The four dominant classes of microstate, labeled A, B, C, and D, are consistently observed in resting-state EEG and can explain 65–84% of the global variance of the data (Michel and Koenig, 2018). The brain dynamic changes can be quantified using many parameters derived from the EEG microstate analysis. Commonly, four temporal parameters are used (Khanna et al., 2015): (1) duration, the mean duration of a microstate class, (2) occurrence, the occurrence frequency per second of a microstate class, (3) coverage, the fraction of total recording time of a microstate, and (4) transition probabilities of a microstate class to any other microstate class. Interestingly, the temporal parameters of microstate classes can represent relative changes in different diseases and in cognitive or behavioral states (Brodbeck et al., 2012; Schumacher et al., 2019; da Cruz et al., 2020).

In the present study, we tested two hypotheses. (1) Reduced brain dynamics, as evidenced by slower microstate dynamic change, is related to the movement disorders of patients with PD. (2) Therapeutic DBS can reverse slow brain dynamics in PD to normal levels.

## Methods

### Participants

Forty-one participants were involved in the study. Twenty were patients with PD (10 female: age range 51–70 with a mean age of 60.2 years; 10 male: age range 50–75 with a mean age of 59.6 years) diagnosed according to the clinical diagnostic criteria of the UK Parkinson's Disease Society Brain Bank. Twenty-one participants were age-matched and gender-matched HCs (seven female: age range 52–58 with a mean age of 55.9 years; 14 male: age range 51–70 with a mean age of 57.8 years). All patients with PD underwent STN-targeted DBS. Clinical assessment was made using the Hoehn and Yahr (H–Y) scale and the Unified Parkinson's Disease Rating Scale III (UPDRS-III) prior to DBS (for baseline) and on the first day of DBS (30 days after surgery). Levodopa equivalent daily dose (LEDD) was calculated for each patient. The inclusion criterion for patients with PD was STN-DBS having a good therapeutic effect. The exclusion criteria were (1) typical PD syndrome induced by drugs or metabolic disorders, encephalitis, or other disease presenting similar symptoms (i.e., multiple system atrophy, progressive supranuclear palsy, and Lewy body dementia), (2) history of significant neurological disease or brain surgery, and (3) neuroimaging findings of severe abnormalities or lesions. The study was approved by the Institutional Review Board of Beijing Tiantan Hospital, Capital Medical University, and written informed consent was obtained from all participants.

### EEG Acquisition and Preprocessing

Before EEG acquisition, all participants were instructed to sit in a comfortable position and relax for 5 min. During recording, participants were instructed to keep their eyes closed and remain awake. Resting-state EEG was recorded using a high-density 256-channel system (EGI System 400; Electrical Geodesics Inc., Eugene, OR, USA). Electrode impedance was kept below 30 kΩ, and 10 min of ongoing EEG data were acquired with a sampling rate of 1,000 Hz. The acquisition reference was Cz. For patients with PD, the EEG acquisition was performed two times. For the first acquisition, the EEG data were recorded before DBS was started (called the DBS-OFF state). For the second acquisition, the EEG recording was performed 24 h after DBS was started (called the DBS-ON state). To eliminate the effects of levodopa-medication, 12 h before the acquisition, patients were asked not to take any anti-PD medications.

To remove muscle artifacts, the electrodes on the face and neck were removed, reducing the system to 204 channels. The EEG data were split into non-overlapping periods of 2 s, and segments contaminated by artifacts were deleted and bad channels were interpolated. To remove the artifact from DBS, which was 130 Hz, a 1–40-Hz band-pass filter was used. Subsequently, independent component analysis was used to remove ballistocardiogram, myoelectricity, and oculomotor artifacts. Thereafter, components related to ballistocardiogram, saccadic eye movements, channel noise, and eye blinking were removed based on the waveform, topography, and spectrogram. Finally, for every participant, 5 min (150 × 2 s periods) of artifact-free EEG data were selected for microstate analysis.

### Microstate Analysis

To estimate the optimal set of topographies that can explain the preprocessed EEG data, we used the atomize and agglomerate hierarchical clustering (AAHC) algorithm to perform standard microstate analysis. First, the EEG signal was further filtered with a bandpass between 2 and 20 Hz (Koenig et al., 2002) and recomputed to obtain an average reference across all channels. The polarity of the topographic maps was ignored in this operation (Pascual-Marqui et al., 1995; Brunet et al., 2011). The global field power (GFP) was calculated, which is the SD of the potentials at all electrodes of an average-reference map (Lehmann and Skrandies, 1980). The GFP represents the electric field strength of the whole brain at every moment, so it is often used to measure the response of the brain to an event or to evaluate the rapid dynamic changes of the brain at the rest state. The local maximum of the GFP curve indicates the moment when the whole-brain electric field is strongest and the topographic signal-to-noise ratios are the highest. In addition, topographic maps tend to be stable during periods of high GFP and then change rapidly to the next topographic map in the GFP minima (Lehmann et al., 1987). Therefore, during the microstate analysis, the topographic maps of the local maximum of the GFP curve can be regarded as discrete states of the EEG signal, and dynamic changes of the EEG signal can be regarded as a variation of these states (Khanna et al., 2015). Afterward, clustering analysis was performed, first to obtain several template maps at the individual level and then across all the participants in each group. To compare vertically and horizontally with other studies, we selected four microstate classes for each group and labeled them A, B, C, and D according to previous studies (Michel and Koenig, 2018). Finally, the spatial correlation between each template map obtained at the group level across all the participants and the topographic map of GFP peaks of the original EEG signal for each individual was calculated. Then, the EEG data at the GFP peak were assigned to the microstate class based on the highest spatial correlation. The microstate of the data point between two GFP peaks was interpolated with the beginning and end segments between two GFP peaks. Three temporal parameters were extracted for each microstate class: (1) microstate duration, (2) microstate occurrence per second, and (3) time coverage of microstate class.

### Data Statistics

For topographic maps of different microstate classes, we performed a comparison between groups with topographical ANOVA (TANOVA) using the Ragu software (https://www.thomaskoenig.ch/index.php/work/ragu/1-ragu) (Habermann et al., 2018). For this, we defined a between-subject design comparing HC and patients with PD with DBS-OFF, and a within-subject design between patients with DBS-OFF and DBS-ON. In addition, a nonparametric randomization test was used on global map dissimilarity. Statistically significant differences were considered when *p* < 0.05.

The differences in microstate duration, occurrence, and coverage among the three groups were calculated using the Statistical Package for the Social Sciences (SPSS v25, IBM Corp., Armonk, NY, USA). To be specific, the differences between HCs and patients with PD with DBS-OFF and patients with PD with DBS-ON were calculated using an independent samples *t*-test. The difference between patients with PD with DBS-OFF and DBS-ON was calculated with a paired-samples *t*-test. The *p*-values < 0.05 were considered statistically significant.

The correlations between temporal parameters of patients with PD with DBS-OFF and UPDRS-III (in DBS-OFF state) and between patients with PD with DBS-ON and UPDRS-III (in DBS-ON state) were calculated using the Pearson's correlation. To explore the relationship between the improvement and temporal parameters of microstates, the correlation between temporal parameters of patients with PD with DBS-ON state and the improvement was calculated using the Pearson's correlation. In addition, the correlation between temporal parameters of patients with PD with DBS-OFF state and LEDD was also calculated using the Pearson's correlation. The *p*-value was corrected by the false discovery rate. The *p*-values < 0.01 were considered statistically significant.

The microstate analysis was carried out with the EEGLAB plugin for microstates (https://www.thomaskoenig.ch/index.php/software/microstates-in-eeglab) in MATLAB 2013b (The MathWorks Inc., Natick, MA, USA).

## Results

### Demographics and Clinical Variables

There were no significant differences in age or gender between patients with PD and HCs. For patients with PD, the illness duration was 8.2 ± 3.6 years, and there were significant differences in the UPDRS-III scale between DBS-OFF state and DBS-ON state (46.5 ± 9.9 vs. 17.1 ± 9.0, *p* < 0.001). The improvement rate was 0.63 ± 0.17.

### Microstate Topographic Characteristics

In previous studies, the four microstate classes were commonly identified in resting-state EEG, and they exhibited high similarity (Michel and Koenig, 2018).

**Figure 1:**
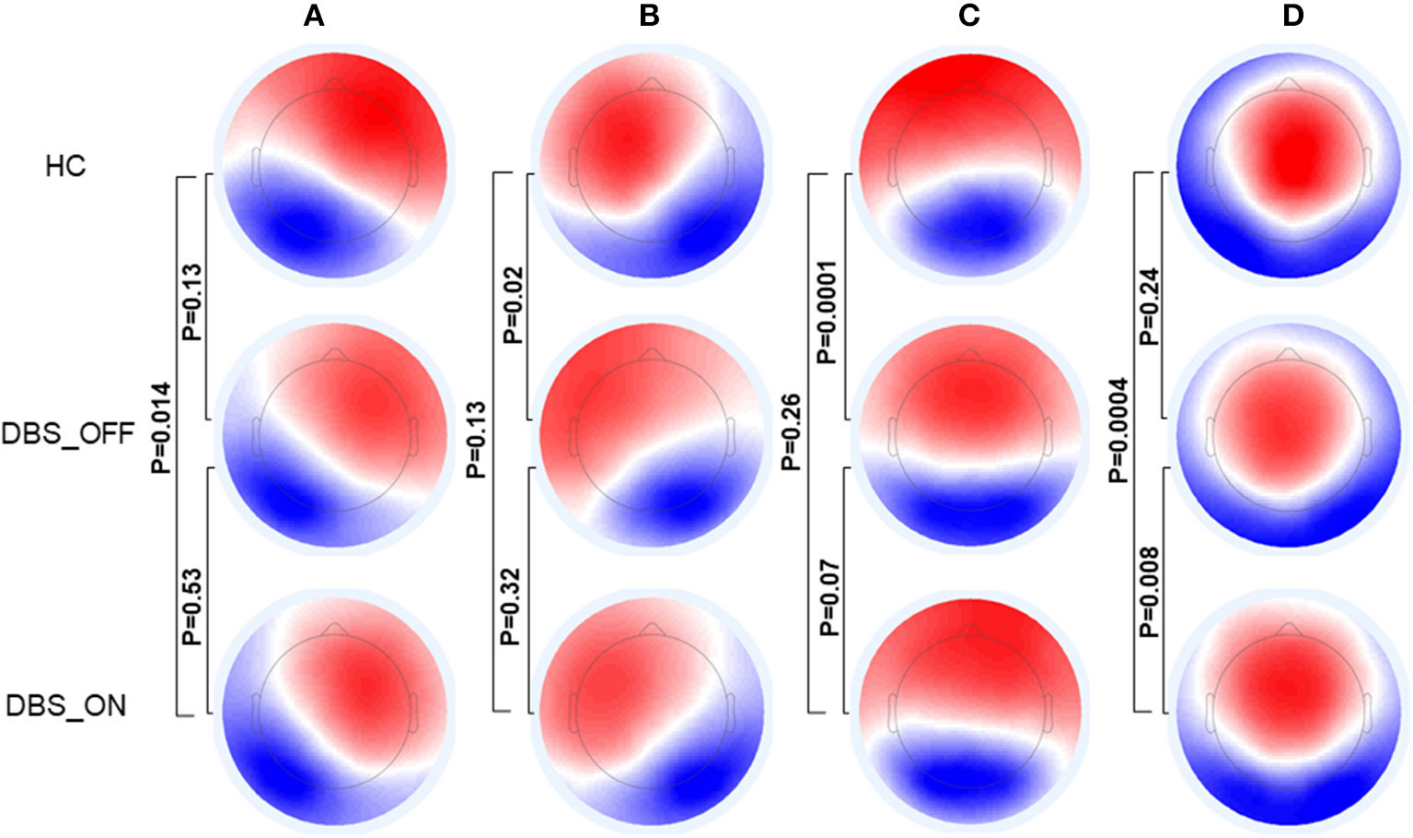
Topographic maps of Microstate **(A–D)**. The *p*-values are yielded by comparing the group topographic maps between groups with TANOVA. DBS, deep brain stimulation; HCs, healthy controls; TANOVA, topographical ANOVA.

Figure 1 shows the microstate classes of the three groups. For HC vs. patients with PD with DBS-OFF state, there were significant differences in microstate class B (*p* = 0.02) and C (*p* = 0.0001). Between HC and patients with PD with DBS-ON state, significant differences were detected in microstate classes A (*p* = 0.014) and D (*p* = 0.0004). However, a difference between patients with PD with DBS-OFF state and DBS-ON state was only reflected in microstate class D (*p* = 0.008).

### Microstate Evaluation and Temporal Characteristics

In the microstate analysis, the four microstate classes explained 80.7% (SD = 2.7%) of the global variance. This was similar to the findings of previous studies (Michel and Koenig, 2018). To be specific, the mean global explained variance was 80.4% (SD = 2.4%) in the HC group, 80.5% (SD = 3.6%) in the patients with PD with DBS-OFF group, and 81.1% (SD = 1.9%) in the patients with PD with DBS-ON group. There were no significant differences between all three groups (*p* = 0.87, *p* = 0.27, and *p* = 0.34).

**Figure 2:**
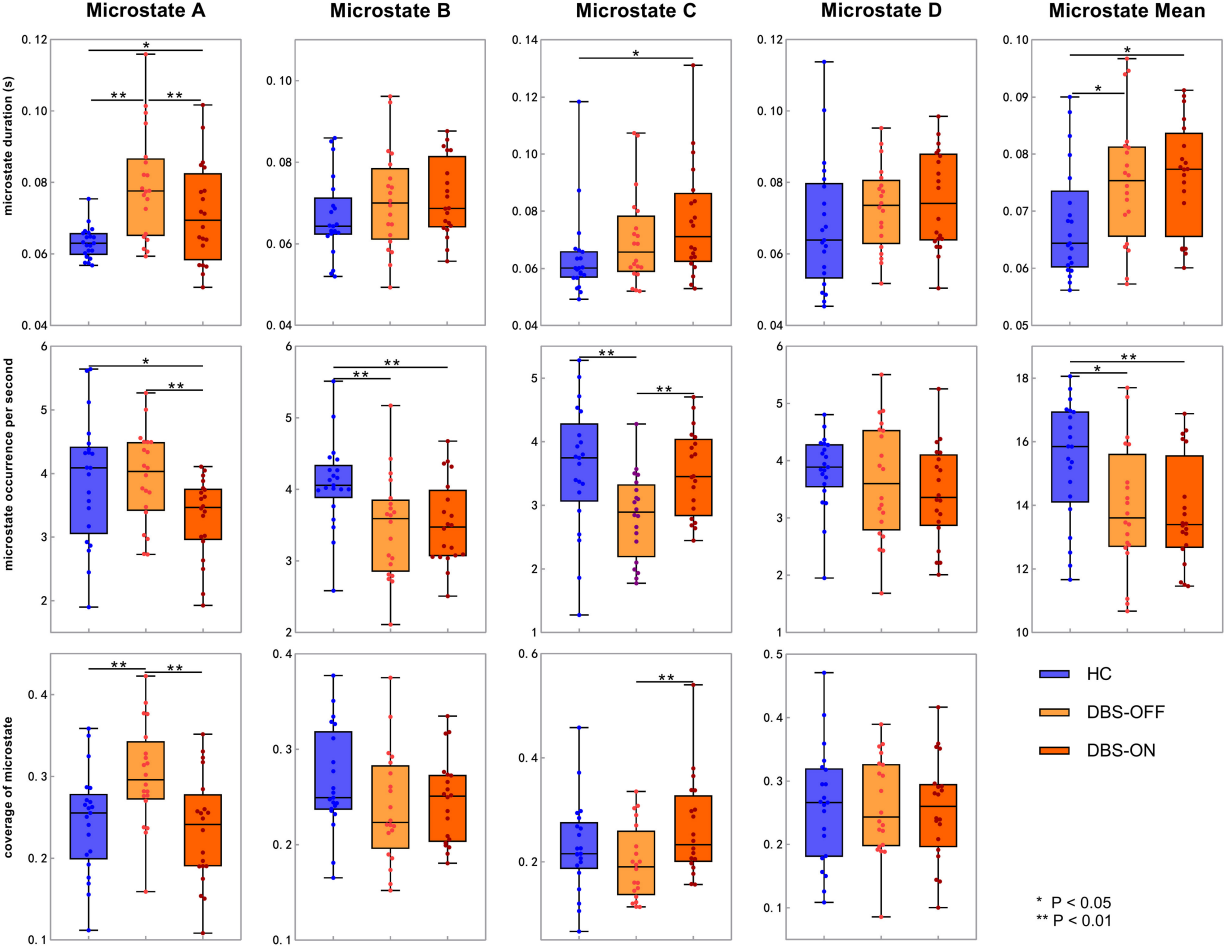
Temporal characteristics of microstate. Between-groups comparison of microstate duration, occurrence per second, and coverage for each microstate class separately. The *p*-values result from the independent samples *t*-test between HCs and patients with PD with DBS-OFF state and DBS-ON state separately and a paired *t*-test between PD patients with DBS-OFF state and DBS-ON state. DBS, deep brain stimulation; HC, healthy controls.

**Figure 3:**
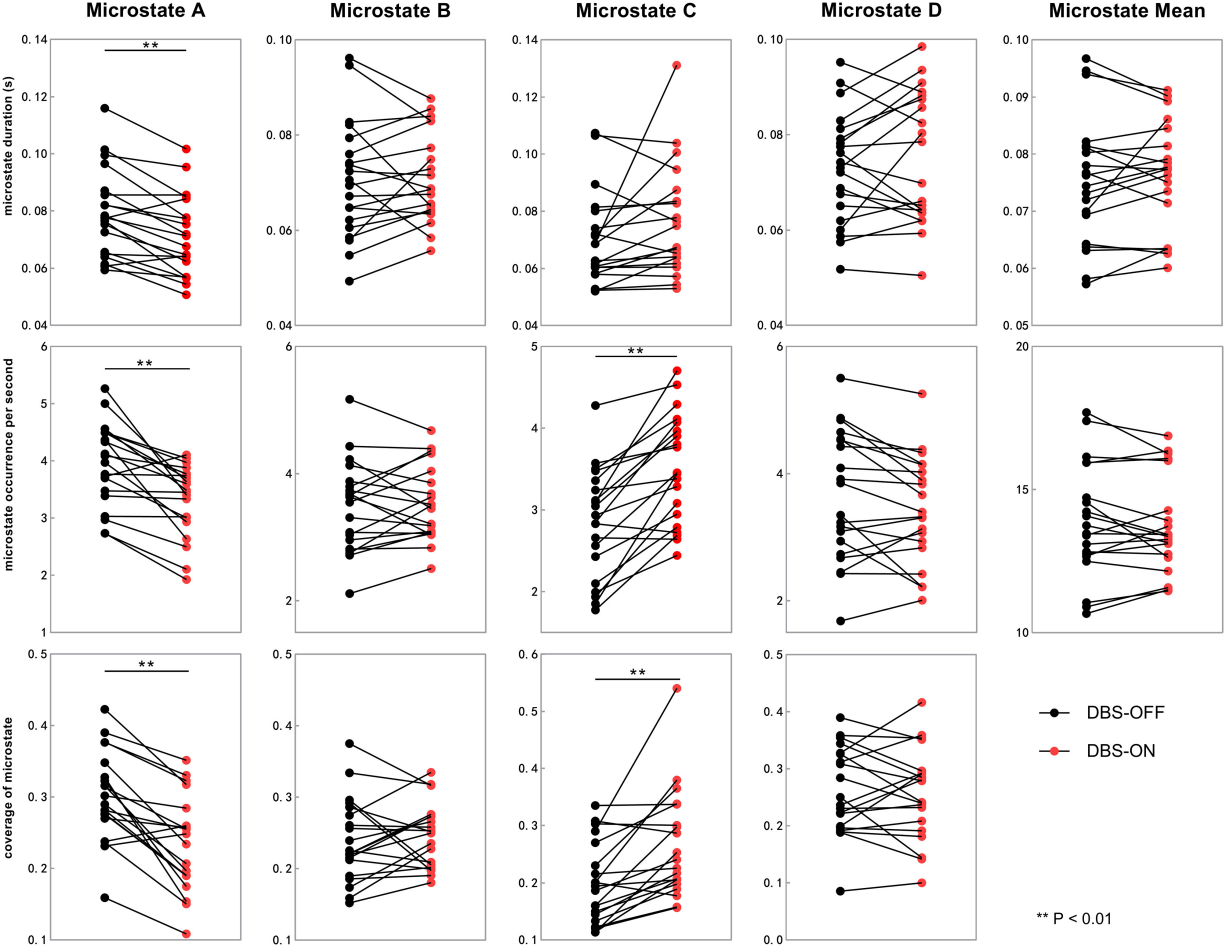
Temporal characteristics of microstate between DBS-OFF state and DBS-ON state. All the microstate temporal parameters decrease after DBS in microstate class A. In addition, the occurrence rate and coverage of microstate class C increase. DBS, deep brain stimulation.

Across all the microstate classes, the mean duration of HCs and patients with PD with DBS-OFF state and DBS-ON state was 0.068, 0.075, and 0.076 s, respectively. The mean duration of the two PD groups was longer than that of the HC group (Figures 2, 3 and Table 1). In addition, the mean microstate occurrence per second was 15.39 in HCs, 13.89 in patients with PD with DBS-OFF state, and 13.77 in patients with PD with DBS-ON state. There were also significant differences between PD groups and HCs (Figures 2, 3 and Table 2).

**Table 1:** Duration of microstate classes A to D and mean of three groups, and results from the comparison between groups with an independent sample *t*-test and a paired sample *t*-test.

	**Healthy controls**	**Patients with PD (DBS-OFF)**	**Patients with PD (DBS-ON)**	**Between-group differences (*****p*****-value)**		
				**HC vs. DBS-OFF**	**HC vs. DBS-ON**	**DBS-ON vs. DBS-OFF**
A	0.063 ± 0.004	0.079 ± 0.015	0.071 ± 0.014	<0.001	0.023	<0.001
B	0.066 ± 0.010	0.071 ± 0.012	0.071 ± 0.010	0.244	0.129	0.758
C	0.064 ± 0.015	0.070 ± 0.016	0.076 ± 0.020	0.214	0.026	0.097
D	0.068 ± 0.018	0.073 ± 0.012	0.073 ± 0.012	0.280	0.157	0.306
Mean	0.068 ± 0.010	0.075 ± 0.011	0.075 ± 0.011	0.031	0.011	0.053

*Values are mean ± SD*.

*DBS, deep brain stimulation; HC, healthy controls; PD, Parkinson's disease*.

**Table 2:** Occurrence per second of microstate classes A to D and mean of three groups, and results from the comparison between groups with an independent samples *t*-test and a paired samples *t*-test.

	**Healthy controls**	**Patients with PD (DBS-OFF)**	**Patients with PD (DBS-ON)**	**Between-group differences (*****p*****-value)**		
				**HC vs. DBS-OFF**	**HC vs. DBS-ON**	**DBS-ON vs. DBS-OFF**
A	3.896 ± 0.985	3.937 ± 0.723	3.313 ± 0.630	0.881	0.031	<0.001
B	4.079 ± 0.597	3.473 ± 0.716	3.524 ± 0.590	0.005	0.005	0.617
C	3.589 ± 1.000	2.831 ± 0.671	3.502 ± 0.671	0.007	0.747	<0.001
D	3.826 ± 0.644	3.649 ± 1.020	3.427 ± 0.849	o.512	0.096	0.052
Mean	15.391 ± 1.877	13.899 ± 1.996	13.766 ± 1.700	0.017	0.006	0.489

*Values are mean ± SD*.

*DBS, deep brain stimulation; HC, healthy controls; PD, Parkinson's disease*.

We also compared the duration, occurrence, and coverage among groups to identify differences in microstate classes A–D. In microstate class A, compared with HCs, the microstate duration of patients with PD increased. However, it decreased after DBS but did not drop back to a healthy level. Correspondingly, compared with HCs and patients with PD with DBS-OFF state, the microstate duration of patients with DBS-ON state decreased. But there was no difference between controls and patients with PD with DBS-OFF state. It was clear that the coverage of microstate class A in patients with PD with DBS-OFF state was significantly higher than that in HCs, but it decreased after DBS dropped back to a healthy level (Figures 2, 3 and Tables 1–3). In microstate class B, there were no differences in duration and coverage between groups. However, the occurrences of the two groups of patients with PD were lower than those of HCs (Figures 2, 3 and Tables 1–3). In microstate class C, after DBS, the duration was longer than that in HCs but not different from before DBS. Compared with controls, the occurrence in patients with DBS-OFF decreased but increased to near control level after DBS. The coverage also increased in patients with PD after DBS (Figures 2, 3 and Tables 1–3). The microstate temporal parameters in microstate class D did not show any differences between any two groups (Figures 2, 3 and Tables 1–3).

**Table 3:** Contribution of microstate classes A to D, and results from comparison between groups with an independent samples *t*-test and a paired samples *t*-test.

	**Healthy controls**	**Patients with PD (DBS-OFF)**	**Patients with PD (DBS-ON)**	**Between-group differences (*****p*****-value)**		
				**HC vs. DBS-OFF**	**HC vs. DBS-ON**	**DBS-ON vs. DBS-OFF**
A	0.244 ± 0.063	0.302 ± 0.062	0.234 ± 0.066	0.005	0.615	<0.001
B	0.267 ± 0.054	0.240 ± 0.058	0.246 ± 0.045	0.126	0.186	<0.565
C	0.229 ± 0.088	0.198 ± 0.071	0.264 ± 0.094	0.218	0.225	<0.001
D	0.260 ± 0.092	0.261 ± 0.078	0.256 ± 0.081	0.971	0.889	0.701

*Values are mean ± SD*.

*DBS, deep brain stimulation; HC, healthy controls; PD, Parkinson's disease*.

### Clinical Correlations

**Figure 4:**
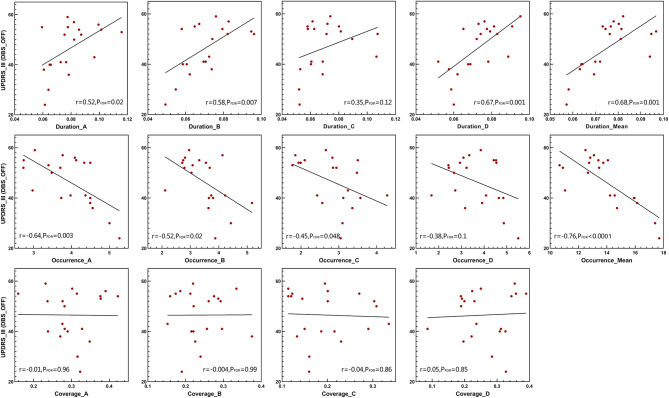
Clinical UPDRS-III correlations. Pearson's correlations between microstate parameters and UPDRS-III scales in DBS-OFF state. DBS, deep brain stimulation; UPDRS-III, the Unified Parkinson's Disease Rating Scale III.

The Pearson correlation was used to test the relationships between several temporal parameters and the UPDRS-III scale. In addition to microstate classes A and C, the microstate duration was positively correlated with the UPDRS-III scale in patients with DBS-OFF (Figure 4). However, this relationship did not exist in patients with DBS-ON. Correspondingly, except for microstate classes B, C, and D, the microstate occurrence per second was significantly negatively correlated with UPDRS-III in patients with DBS-OFF (Figure 4). There were also no relationships between occurrence rates and UPDRS-III in patients with DBS-ON. In addition, there was no relationship between microstate coverage and UPDRS-III in patients with PD with DBS-OFF state or DBS-ON state (Figure 4). We also explored the correlation between improvement rate and microstate temporal parameters; however, we found no relationships between them. There were also no relationships between microstate temporal parameters and LEDD.

## Discussion

In this study, we used the EEG microstate analysis in HCs and patients with PD with DBS-OFF state and with DBS-ON state to demonstrate the effects of PD and therapeutic DBS on dynamic changes in the brain.

### Microstate Dynamics of Patients With PD

We found an obvious decrease in brain dynamic changes in patients with PD compared with HCs. Overall, patients with PD showed a longer mean microstate duration and fewer occurrences per second than HCs. The EEG microstate can be an indicator of brain variability and can show elaborate dynamic properties that are important for optimal brain function (Schumacher et al., 2019). The microstate sequences exhibit scale-free or fractal dynamics in a healthy brain (Van de Ville et al., 2010). Such scale-free properties are vital for brain dynamics to respond to incoming information. In microstate analysis, duration is the most crucial parameter, because precise timing is vital for the brain to deal with the constant flow of information (Van de Ville et al., 2010). The prolonged duration observed in patients with PD damaged the intricate fractal properties. Our results are in line with those of previous studies. Schumacher et al. (2019) examined the brain dynamic changes of Lewy body dementia (including PD dementia) using the EEG microstate analysis and found obvious decreases compared with controls. Coincidentally, Kim et al. (2017) demonstrated two discrete connectivity configurations: a more frequent state (State I) and a less frequent state (State II) by dynamic functional connectivity analyses. State I decreased by 12.62% and State II increased by the same amount in patients with PD. However, brain function requires efficient reconfiguration of activity. This flexibility is crucial for the coordinated engagement of brain regions to accomplish various activities. Impaired brain flexibility can lead to aberrant brain function. PD is a disabling neurodegenerative disease characterized by reduced ability to move and slowing of cognitive processes. Such degenerative changes in PD may derive from a decline in the flexibility of the brain (Sorrentino et al., 2021). In our results, the longer mean duration of a microstate is parallel to the longer mean dwell time and fewer occurrences in each microstate, which means reduced flexibility of the brain. This notion is supported by a study that demonstrated reduced global brain network efficiency and a more random network organization in PD (Olde Dubbelink et al., 2014).

Furthermore, in agreement with previous studies (Kim et al., 2017; Schumacher et al., 2019; Sorrentino et al., 2021), we also found that the reduced brain dynamic changes were significantly associated with clinical symptoms, indicating that longer mean duration correlated with a more severe movement disorder. In addition, we found the occurrences per second of microstates were negatively correlated with neural function, in agreement with former results. This means that the reduced reconfiguration rate of brain activity is parallel to inefficient information processing. Except for the duration of microstate classes A and C and the occurrence of microstate classes B, C, and D, other parameters were wholly correlated with the UPDRS-III scale, which suggests that the effects of microstate changes on impaired movement are universal and widespread rather than inclined to one or two absolute microstates. However, the microstate coverage was not associated with neural function, which further highlights that dwell time and occurrence of microstates are the main parameters of brain dynamic changes.

### DBS Effects on Microstate Dynamics in PD

Deep brain stimulation has extensive impacts on the global brain network of patients with PD (Kahan et al., 2012, 2014; van Hartevelt et al., 2014). DBS can reshape large-scale brain networks impaired by PD back toward normal function (Kahan et al., 2012, 2014; van Hartevelt et al., 2014; Deco et al., 2015). Saenger et al. (2017) used resting-state fMRI and whole-brain computational modeling to explore the effects of DBS on brain dynamic changes. They found that brain dynamic activity was more stereotyped and less flexible in PD, whereas DBS created a more flexible state, which was shown by higher phase consistency in patients with DBS-ON state than in patients with DBS-OFF state. In this study, although the microstate mean duration and mean occurrence per second were not significantly different, the coverage of microstate classes A and C showed obvious changes, and the variation tendency was dropped back toward a healthy state in patients with PD with DBS-ON state compared with patients with PD with DBS-OFF state. Our EEG microstate analysis results are not only consistent with commonly used analysis methods (such as fMRI and magnetoencephalography) but also open new avenues to study the effects of DBS on large-scale brain dynamic change. In the present study, the mean microstate duration and occurrence per second were not different between DBS-ON state and DBS-OFF state, and both had similar differences with HCs. The reason for this may be that EEG recording of patients with PD with DBS-ON state was made 24 h after the initiation of DBS, which might not be enough time for the brain to produce significant network changes. This study focused on the therapeutic effect of acute DBS stimulation, and it is well-known that large-scale brain network changes are the result of long-term effects. Okun (2012) described DBS inhibiting cells and exciting fibers (Vitek, 2002; McIntyre and Hahn, 2010) around the electrode, which changed the firing rate and pattern of individual neurons in the base ganglia (Wichmann et al., 2011). DBS also acts as synapses and triggers neighboring astrocytes to promote the release of calcium and neurotransmitters; furthermore, it can cause local increase in cerebral blood flow (Lee et al., 2004, 2011; Tawfik et al., 2010; Vedam-Mai et al., 2012). Finally, DBS induces local and possibly distal proliferation of neural precursor cells. The long-term effects of these actions will eventually lead to large-scale network changes. In the present study, although the symptoms of patients with PD in the DBS-ON state were significantly improved compared with those in the DBS-OFF state and several microstate parameters were changed after DBS, the effect of acute DBS on the whole-brain network was limited. Therefore, the effect of acute DBS stimulation on changing the whole-brain dynamic was limited. We only observed a difference before and after stimulation in individual microstates, but there was no obvious difference in the overall mean value. However, previous studies have demonstrated that microstate classes A, B, C, and D represent different brain network configurations. It is difficult for acute DBS to fully activate multiple networks at the same time to change whole-brain dynamics. Although acute DBS changed individual microstates, these changes were not sufficient to significantly change the mean values of all microstates. In addition, there are were no differences between the UPDRS-III scale and microstate parameters or between improvement and microstate parameters in patients with DBS-ON. Such results may be derived because DBS confers acute phase effects rather than long-term effects. Previous studies have mainly focused on brain activity for 6 months or longer after DSB. We, therefore, suggest that further work is required to reveal the effects of long-term DBS on microstate changes.

### Limitations

There are some limitations to this study. First, the sample size was relatively small, and a larger test population may yield more significant and robust results. In addition, we did not consider the relationship between microstate changes and the cognitive level. Dementia is one of the most common and important non-motor symptoms encountered in advanced PD (Aarsland et al., 2017). Prolonged microstate duration occurs in Lewy body dementia (including PD dementia) and is significantly associated with the severity of cognitive impairment (Schumacher et al., 2019). Finally, the EEG acquisition was performed 24 h after the initiation of DBS. The action time of DBS is relatively short, so the effect of DBS is not in a stable state. Many studies that explored the effects of DBS on brain networks in PD focused on long-term results (McIntyre and Hahn, 2010; Litvak et al., 2011; Akram et al., 2017; Horn et al., 2017, 2019). Long-term action of DBS may lead to a more stable and obvious effect on the brain. In addition, a large-scale change in brain networks is a chronic process that may induce local and possibly distal proliferation of neural precursor cells (Okun, 2012).

## Conclusions

Resting-state EEG microstate analysis can detect sub-second timescale brain dynamics of the brain in PD and is an important tool for the exploration of DBS effects on large-scale brain networks. Slowing of brain dynamics is a prominent feature in patients with PD and is significantly associated with neural impairments. Therapeutic acute DBS can partially revert brain dynamics toward a healthy level.

## Data Availability Statement

The raw data supporting the conclusions of this article will be made available by the authors, without undue reservation.

## Ethics Statement

The studies involving human participants were reviewed and approved by the Institutional Review Board of Beijing Tiantan Hospital. The patients/participants provided their written informed consent to participate in this study.

## Author Contributions

FM, QuW, and ZL: conception and design. ZL, CL, KL, and QiW: acquisition of data. CH and ZL: analysis and interpretation of data. ZL: drafting the article and reviewed submitted version of manuscript. HQ and FM: critically revising the article. GR and ZL: statistical analysis. GR and QuW: administrative/technical/material support. JZ: study supervision. All authors contributed to the article and approved the submitted version.

## Conflict of Interest

The authors declare that the research was conducted in the absence of any commercial or financial relationships that could be construed as a potential conflict of interest.
